# Crosstalk between Erythropoiesis and Iron Metabolism

**DOI:** 10.1155/2010/317095

**Published:** 2010-08-24

**Authors:** Stefano Rivella, Elizabeta Nemeth, Jeffery Lynn Miller

**Affiliations:** ^1^Division of Hematology-Oncology, Department of Pediatrics, Weill Cornell medical College, New York, NY 10021, USA; ^2^David Geffen School of Medicine at UCLA, CHS 37-131, 10833 Le Conte Avenue, Los Angeles, CA 90095-1690, USA; ^3^Molecular Medicine Branch, NIDDK, NIH, Bethesda, MD 20892, USA

Oxygen delivery is essential to sustain all vital functions. Vertebrate organisms have, therefore, evolved sophisticated and tightly regulated mechanisms to ensure continuous red cell synthesis. Chronic or acute blood losses trigger multiple feedback mechanisms aimed at increasing erythropoiesis and minimizing the damage associated with profound anemia and hypoxia. 

Iron is an element of paramount importance for erythropoiesis, being an essential part of the hemoglobin metalloprotein that ensures oxygen absorption, transport, and delivery. Therefore, it is not surprising that iron absorption and transport are closely linked to the demands of erythropoiesis. However, the mechanisms underlying this interconnected relationship have only recently started being understood. 

The focus of this special issue is on iron metabolism, erythropoiesis, and their close association, with a special emphasis on proteins regulating iron metabolism and how erythropoiesis affects their expression. The mechanistic view will be integrated with the clinical observations in disease such as myelodysplastic syndromes, sickle cell anemia, Diamond-Blackfan anemia, and anemia of inflammation. And finally, in light of these new discoveries, new potential therapies for well-known disorders such as beta-thalassemia will be discussed. 

In the last decade, new discoveries have fueled the field of iron metabolism. The papers by H. Li and Y. Z. Ginzburg[1],E. Nemeth [2], L. Cianetti et al. [3], and T. Tanno and J. L. Miller [4] provide a comprehensive and updated overview of the proteins involved in regulating dietary iron absorption, plasma iron concentrations, tissue iron distribution, and erythropoiesis. The review by H. Li and Y. Z. Ginzburg [1] introduces the mechanisms of crosstalk between iron and erythropoiesis and their function in different diseases. E. Nemeth [2] describes the role of the peptide hormone hepcidin in iron physiology and in many iron-related disorders and highlights the potential of hepcidin agonists and antagonist as future therapeutic tools for iron disorders. The paper by L. Cianetti et al. [3] describes the hepcidin receptor and iron transporter ferroportin and its role during normal and pathological erythroid differentiation. The paper by T. Tanno and J. L. Miller [4] describes the relationship between iron regulation and ineffective erythropoiesis, focusing on potential mechanisms for pathological iron overloading.

Improved knowledge on proteins that interconnect iron metabolism and erythropoiesis is opening new prospective to develop new therapies, such as in beta-thalassemia. The review by L. Melchiori et al. [5] provides important evidence that use of Jak2 inhibitors or hepcidin agonists may ameliorate the abnormal erythropoiesis and iron overload in *β*-thalassemia. 

The interdependence of erythropoiesis and iron regulation extends beyond normal metabolism to include several pathological conditions. Diseases that are primarily associated with erythroid defects often lead to iron overload, maldistribution, or deficiency. The reviews by E. Messa et al. [6] and by M. A. Badawi et al. [7] summarize the historical progress and recent clinical developments using iron chelation in patients with myelodysplastic syndromes. Similarly, the paper by R. Raghupathy et al. [8] provides an overview of transfusional iron overload patients with sickle cell disease, highlighting how to prevent iron overload in this disorder. 

Diamond-Blackfan anemia is a rare congenital pure red-cell aplasia that presents during infancy. D. Chiabrando and E. Tolosano's [9] review focused upon heme and ribosomal biogenesis in this disease and why deficiency of the heme exporter Feline Leukemia Virus subgroup C Receptor (FLVCR1) may cause a related phenotype.

Finally, the review by E. A. Price and S. L. Schrier [10] provides a comprehensive and updated overview of anemia of inflammation, focusing on many of key clinical issues that remain to be clarified, such as understanding of mechanisms of anemia of inflammation in “noninflammatory” diseases, optimal methods for distinguishing anemia of inflammation from iron deficiency anemia, and understanding the contributory role of various pathologic mechanisms in individual human diseases that lead to anemia of inflammation. 

We hope that this special issue will stimulate curiosity, interest, and new research directions in exploring the field of iron and erythropoiesis, with the ultimate goal to develop new drugs and improve the management of disorders associated with altered iron metabolism, abnormal erythropoiesis, and anemia of inflammation. 

*Stefano Rivella* *Elizabeta Nemeth* *Jeffery Lynn Miller*
